# The R203W substitution drives PACS‐1 syndrome by disrupting intramolecular regulation

**DOI:** 10.1111/febs.70492

**Published:** 2026-03-20

**Authors:** Troy C. Krzysiak, In‐Ja L. Byeon, Ryan Ponticelli, Mary E. Lucas, Lariah Thompson, Christopher DeHaven, Gary Thomas, Angela M. Gronenborn

**Affiliations:** ^1^ Department of Structural Biology University of Pittsburgh School of Medicine Pittsburgh PA USA; ^2^ Department of Microbiology and Molecular Genetics University of Pittsburgh School of Medicine Pittsburgh PA USA; ^3^ Hillman Cancer Center University of Pittsburgh School of Medicine Pittsburgh PA USA

**Keywords:** HDAC6, NMR, PACS‐1, PACS‐2, rare diseases

## Abstract

The c607C>T mutation in the *PACS1* gene results in an Arg203Trp substitution in the multifunctional protein PACS‐1, and drives a syndrome characterized by intellectual disability, seizures, craniofacial dysmorphisms, and various characteristics of the autism spectrum. On the molecular level, this syndrome, in part, results from enhanced association of PACS‐1 with the protein deacetylase HDAC6. PACS‐1 uses its Furin binding region (FBR: amino acids 101–273) to directly interact with the catalytic domains of HDAC6. We present the solution structure of a chimeric PACS‐1 FBR and use NMR to demonstrate that the PACS‐1/HDAC6 interaction is regulated by an intramolecular mechanism involving the central unstructured region of PACS‐1 folding back across the FBR and engaging in contacts with an extended, positively charged loop. The R203W substitution, located in this loop, disrupts this regulatory interaction and, *in vitro*, displays the ability to promote aberrant protein–protein interactions.

## Graphical abstract

The middle region (MR) of PACS‐1 controls engagement with specific partner proteins. This manuscript presents the structure of the Furin binding region (FBR) and how interactions with partners are regulated through the interplay between a basic patch in the FBR and an acidic cluster in the MR. PACS‐1 syndrome, a neurological disorder caused by the R203W substitution in the FBR, disrupts this intramolecular interaction and may promote aberrant protein–protein interactions.

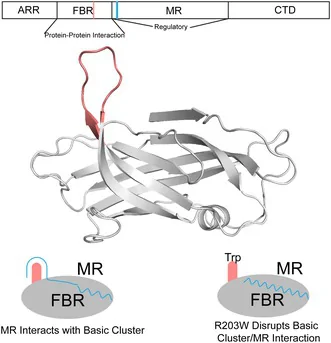

**Abbreviations**
AF: AlphaFold
BRCT: BRCA1 C‐terminal domain
CD: circular dichroism
FBR: Furin binding region
FHA: Forkhead associated domain
GGA3: Golgi‐localized, gamma adaptin ear‐containing 3, ARF‐binding family
HDAC6: histone deacetylase 6
HEPES: 2‐[4‐(2‐hydroxyethyl)piperazine‐1‐yl]ethanesulfonic acid
HSQC: heteronuclear single quantum coherence
MAM: mitochondria associated membrane
MBP: maltose binding protein
MR: middle region of PACS proteins
Nef: negative regulatory factor
NOESY: nuclear overhauser effect spectroscopy
PACS: phosphofurin acidic cluster sorting protein
PKD2: polycystic kidney disease 2
SEC‐MALS: size exclusion multi‐angle light scattering
SIRT1: silent mating type information regulation two homologue 1
TCEP: tris(2‐carboxyethyl)phosphine
TOCSY: total correlation spectroscopy
TROSY: transverse relaxation‐optimized spectroscopy
WW: tryptophan‐tryptophan domain

## Introduction

Phosphofurin acidic cluster sorting (PACS‐1) is a broadly expressed, multifunctional protein with roles in protein and membrane trafficking [1, 2, 3, 4, 5], HIV immune evasion [6, 7], genome stability [8], and neurobiology [9, 10, 11]. Genome‐wide association studies (GWAS) suggest polymorphisms in *PACS1* contribute to severe childhood obesity and bipolar disorder [12, 13]. Recently, a recurrent missense mutation in *PACS1* (c607C>T) that results in an Arg203Trp (R203W) substitution has been identified as the causative agent of a severe neurological disorder known as PACS‐1 syndrome or Schuurs–Hoeijmakers syndrome [9]. Individuals carrying this mutation exhibit characteristic craniofacial dysmorphisms and several neurological abnormalities including: developmental delay, intellectual disability with varying degrees of autism, and seizures [9, 14, 15, 16, 17]. Mechanistically, this syndrome is poorly understood.

Much of the 963 amino acid PACS‐1 protein is unstructured. There are two large unstructured regions; at the N‐terminus resides the ill‐structured atrophin‐1‐related region (ARR), named for its sequence similarity with the nuclear transcriptional corepressor [18]. The second, the middle region (MR), regulates PACS‐1 protein–protein interactions and separates the protein's two structured domains: the Furin binding region (FBR) and a C‐terminal PACS domain (CTD) of unknown fold and function [19]. The FBR is the most extensively studied region of the protein given that it is essential for PACS‐1 interaction with sorting adapters such as AP‐1, AP‐3, and GGA3, as well as cellular cargo [2, 20]. PACS‐1 protein–protein interactions often occur through recognition of an acidic cluster in the interacting partner [1, 5, 21], though, increasing evidence indicates that the complete PACS‐1 recognition site on a partner is more complex [21, 22]. The pathogenicity of an amino acid substitution commonly results from substitution‐induced changes in the protein's structure or dynamics, or from alterations to important protein–protein interactions [23, 24, 25]. The PACS‐1 R203W substitution is located in the FBR, positioned in a cluster of basic amino acids (^196^RRKRYKNR^203^), which might be important for recognition of acidic patches in potential partners. Mutagenesis of this cluster has implicated a role for it in PACS‐1 interaction with CK2*β* and various HDACs [8, 20], but direct evidence of binding between the PACS‐1 basic cluster and a partner acidic cluster has not been reported. Nevertheless, the R203W substitution has the potential to drive pathogenesis through each common mechanism. Interestingly, charge‐altering substitutions in the PACS‐1 paralog, PACS‐2, were reported as the causative agents of another neurological disorder with phenotypes similar to those of PACS‐1 syndrome [26, 27], highlighting the importance of charge–charge interactions in PACS protein function.

HDAC6 is a class II histone deacetylase responsible for the deacetylation of α‐tubulin lysine 40 [28, 29] which, when acetylated, promotes microtubule stability [30]. Villar‐Pazos *et al*. found that neurons, as well as patient‐derived fibroblasts expressing PACS‐1 containing the R203W substitution, exhibited increased immunoprecipitation of PACS‐1 R203W with HDAC6, profoundly disrupted cytoskeletal architecture, and a dispersed Golgi [10]. Importantly, Villar‐Pazos *et al*. demonstrated that silencing HDAC6 could rescue PACS‐1 syndrome cellular phenotypes, strongly suggesting that the relationship between PACS‐1 and HDAC6 is critical for disease manifestation [10]. While both the PACS‐1 FBR and CTD contribute to PACS‐1/HDAC6 association, current evidence suggests that the FBR drives the association between the two proteins, similar to its role in cellular trafficking interactions [10].

In this manuscript, we demonstrate that the PACS‐1 FBR is capable of directly contacting the two catalytic domains of HDAC6 and that this communication is modulated by an acidic cluster immediately C‐terminus to the FBR that interacts with the basic cluster of the FBR. We also present the NMR solution structure of the FBR which reveals that the FBR exhibits a C2 fold (similar to the second domain of Ca^2+^‐dependent protein kinase C) with the basic cluster existing as a positively charged loop protruding away from the core *β*‐sandwich structure. Finally, we demonstrate that the R203W substitution disrupts PACS‐1 intramolecular regulation and has the ability to promote aberrant protein–protein interactions.

## Results

### The PACS‐1 FBR and HDAC6 catalytic domains physically interact

The association between PACS‐1 and HDAC6, first identified by Villar‐Pazos *et al*., was based on co‐immunoprecipitation experiments that do not require direct physical interaction between two proteins for identifying a functional relationship [10]. Therefore, to verify that a direct physical interaction between these two proteins exists, ^1^H^15^N HSQC spectroscopy was employed. Spectra of uniformly ^15^N‐labeled (^15^N)‐PACS1^101‐273^ (FBR) were recorded in the absence and presence of natural abundance (na)‐HDAC6 catalytic domain 1 (CD1) or 2 (CD2). Addition of the ~40 kDa CD1 to the FBR resulted in extensive line‐broadening of amide resonances of the FBR spectrum, with only resonances of very flexible, unstructured regions remaining (Fig. 1A). This unambiguously demonstrates that a direct physical interaction between the FBR and CD1 occurs. Addition of an equal amount of the identically sized CD2 to the FBR also resulted in broadening of FBR resonances (Fig. 1B) establishing that both catalytic domains are capable of engaging the PACS‐1 FBR. Qualitatively, more pronounced effects on the FBR ^1^H^15^N HSQC spectrum were observed in the presence of CD1 suggesting FBR/CD1 binding is tighter than that of FBR/CD2, in agreement with the report by Villar‐Pazos *et al*. [10].

**Fig. 1:**
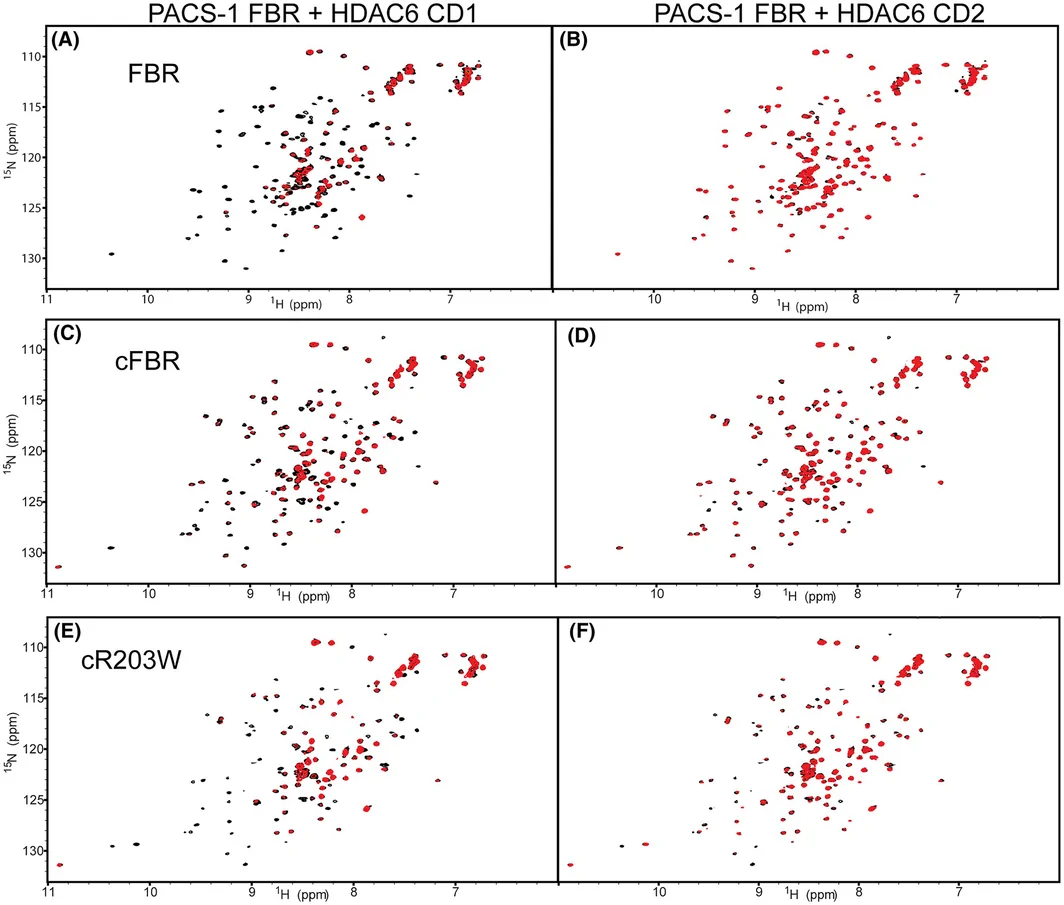
The PACS‐1 FBR physically interacts with both HDAC6 Catalytic Domains. ^1^H^15^N HSQC spectra of the 30 μm samples of PACS‐1 (A,B) FBR, (C,D) cFBR, or (E,F) cR203W in the absence (black) or presence (red) of (A,C,E) 60 μm HDAC6 catalytic domain (CD) 1 or (B,D,F) catalytic domain 2. Spectra were collected at pH 6.5, 30 °C, 800 MHz.

We next aimed to characterize the effect of the R203W substitution on the PACS‐1 FBR/HDAC6 CD1/2 interaction. Unfortunately, while the R203W substituted FBR was expressed at comparable levels to the wild‐type protein, it accumulated in inclusion bodies. Attempts to refold the R203W‐substituted FBR were unsuccessful, and even extensive trials to obtain sufficient amounts of soluble, folded protein for structural studies failed. This prompted us to adopt a more targeted approach relying on the solution properties of the PACS‐1 ortholog, PACS‐2 [22]. The ^1^H^15^N HSQC spectrum of wild‐type PACS‐1 FBR exhibits reasonable quality only at low protein concentrations (30 μm), with spectral quality diminishing as protein concentration is increased, unlike the PACS‐2 FBR (Fig. S1A–D and [22]), despite the two domains containing only 34 amino acid differences (Fig. S2). We hypothesized that substitution of a few PACS‐2 amino acids into their corresponding positions in the PACS‐1 FBR might generate a chimeric FBR with more favorable biophysical properties to allow for the expression of a more soluble protein and more straightforward purification of the R203W substituted PACS‐1 FBR. Substitution of only three PACS‐1 FBR amino acids with the corresponding PACS‐2 amino acids (P244K, K249W, and Y251A) resulted in a substantially better‐behaved protein at the concentrations necessary for structure determination (Fig. S1E,F). This chimeric FBR (cFBR) exhibited interactions with the HDAC6 CD1 comparable to those of the wild‐type FBR (Fig. 1C,D). Furthermore, introduction of the R203W substitution into the cFBR (cR203W) resulted in soluble protein that also displayed comparable interaction with HDAC6 CDs akin to those of both the wild‐type and chimeric FBRs (Fig. 1E,F). However, the substitution resulted in increased protein self‐interaction as evidenced by spectral quality similar to observations with wild‐type PACS‐1 FBR (Fig. S1G,H).

### The R203W substitution does not reduce the thermal stability of the PACS‐1 FBR

Since the R203W substitution made the wild‐type PACS‐1 FBR refractory to protein purification, we postulated that the amino acid change might reduce the thermal stability of the domain. Numerous down‐field resonances, distinct from those located in the random coil region of 8.1–8.4 p.p.m., in the ^1^H^15^N HSQC spectra of PACS FBRs, indicated that these domains contain mostly *β*‐sheet secondary structure. We, therefore, performed circular dichroism measurements at 220 nm to monitor thermal unfolding [31, 32]. The PACS‐1 and PACS‐2 wild‐type FBRs exhibited *T*_m_ values of ~54 °C and 57 °C, respectively (Fig. 2). As suggested by the improved quality of the ^1^H^15^N HSQC spectrum, the cFBR, was stabilized by ~19 °C compared to the wild‐type PACS‐1 FBR, exhibiting a *T*_m_ of ~70 °C. Increased thermal stability was also observed for cR203W, which exhibited a *T*_m_ value of ~68 °C (Fig. 2). All FBRs precipitated upon thermal unfolding.

**Fig. 2:**
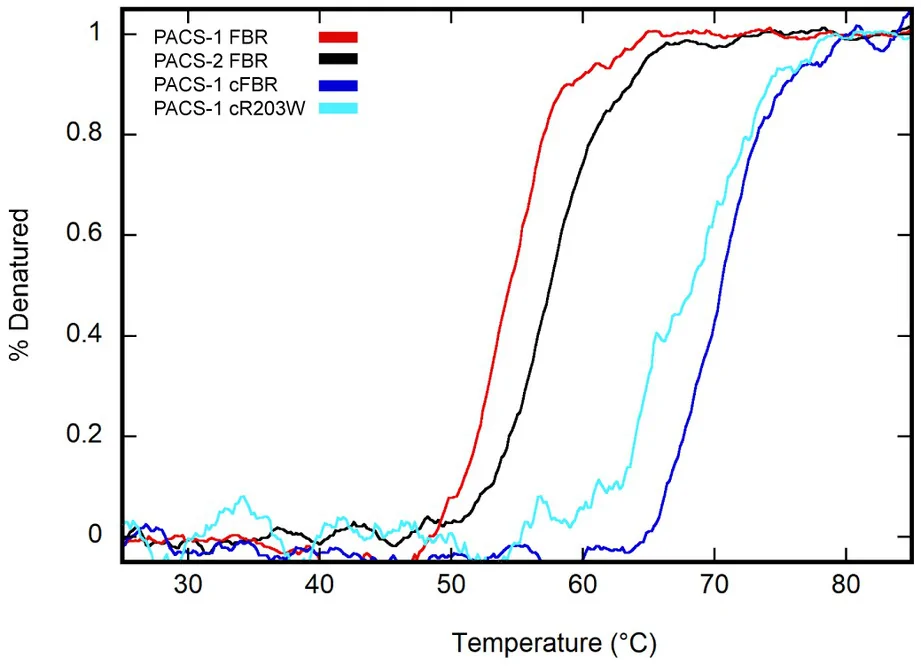
The R203W substitution does not alter FBR stability. Superposition of thermal denaturation curves for the PACS‐1 FBR (red), PACS‐2 FBR (black), PACS‐1 cFBR (blue), and PACS‐1 cR203W (cyan). Molar ellipticity at 220 nm was measured over the temperature range 20–85 °C. The observed *T*_m_ values are 54 °C, 57 °C, 70 °C, and 68 °C, respectively.

### NMR structure of the PACS‐1 FBR

AlphaFold (AF) predictions suggest the (^196^RRKRYKNR^203^) basic cluster has a few residues at the end of a *β*‐strand but mostly occupies a large loop on one side of the FBR [33, 34]. Introduction of a large hydrophobic side chain into an exposed loop such as this, potentially, can generate new intermolecular protein–protein interactions. Such induced intermolecular protein associations may account for the observed decrease in the cR203W ^1^H^15^N HSQC spectrum quality at higher protein concentration. To verify the computational predictions and because no experimentally derived structures for any part of the PACS proteins are available to date, we determined the solution structure of the PACS‐1 cFBR using NMR spectroscopy.

Overall, assignment of more than 95% of all backbone and side chain resonances of the cFBR, comprising residues 101 through 273, and the cFBR294, comprising residues 101 through 294, was achieved. Importantly, the additional amino acids in the cFBR294 contain critical autoregulatory components of the MR [20]. NMR structure calculations were performed using XPLOR‐NIH [35] with 4689 NOE‐derived distance constraints and 279 dihedral angle constraints based on HN, N, C*α*, and C*β* chemical shifts using TALOS‐N [36]. The lowest energy 40‐conformer ensemble (Fig. 3A) satisfies all experimental constraints and displays excellent covalent geometry with atomic root‐mean‐square differences (r.m.s.d.) of 0.57 ± 0.05 and 1.07 ± 0.06 Å for the backbone and all heavy atoms, respectively, relative to their mean positions (Table 1). The largest r.m.s.d. values (> 3 Å) were observed for the unstructured C‐terminus (residues 260–273) and the first loop (residues 110–113) due to a low number of experimental constraints (Fig. 3A).

**Fig. 3:**
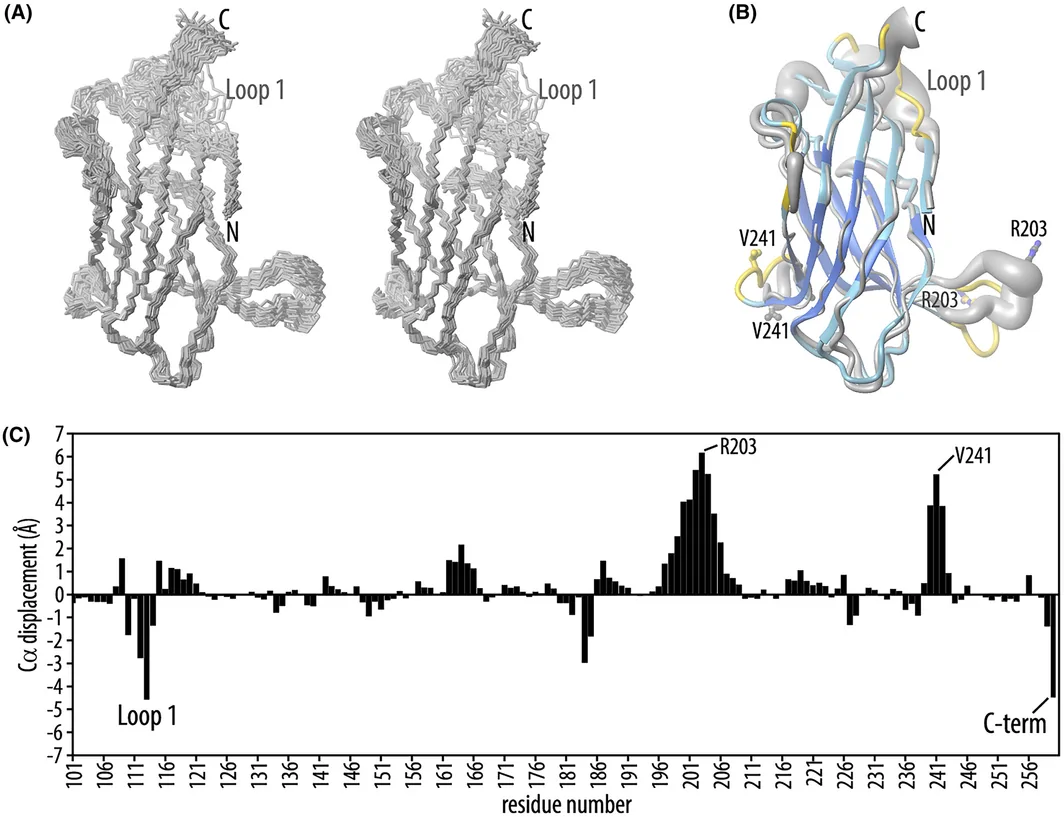
Solution structure of the PACS‐1 cFBR. (A) Stereo‐view of a superposition of the final 40 conformers (backbone N, C*α*, C′ atoms) of the NMR structure of the PACS‐1 cFBR. The N‐terminal and C‐terminal ends of the chain are labeled with ‘N’ and ‘C’, respectively, and the flexible first loop is labeled Loop 1. (B) Superposition of a worm model of the experimental NMR structure (gray) and the AF2 ribbon model (blue and gold). The thickness of the worm is proportional to the pairwise Cα r.m.s.d. values, and the AF2 model is colored according to confidence levels: highly confident, royal blue; confident, sky blue; low confidence, gold. Sidechains of amino acids exhibiting the largest structural differences, R203 and V241, are shown in stick and ball representation. The flexible C‐terminal tail (residues 261–273) is not included in A and B for clarity reasons. Structures were generated using MOLMOL v.2 K.2 [37]. (C) Bar graph depicting Cα coordinate differences between the mean NMR structure and the AF2 model along the chain where normalized values were obtained by subtracting the average C*α* coordinate differences (plus 1 Stdev) between the 40 conformers in the NMR ensemble from the C*α* coordinate differences between the mean NMR structure and model (Fig. S3).

**Table 1:** Statistics for the final 40 conformer ensemble of the PACS1^101‐273^.

Number of distance constraints	
Intra‐residue (i−j = 0)	1664
Sequential (|i−j| = 1)	1010
Medium range (2 ≤ |i−j| ≤ 4)	362
Long range (|i−j| ≥ 5)	1509
H‐bonds	144
Total	4689
Number of dihedral angle constraints	
φ	140
ψ	139
Total	279
Structural quality	
Violations^a^	
Distance constraints (Å)	0.026 ± 0.001
Dihedral angle constraints (deg)	0.497 ± 0.095
Deviation from idealized covalent geometry	
Bond lengths (Å)	0.005 ± 0.000
Bond angles (deg)	0.611 ± 0.009
Improper torsions (deg)	0.418 ± 0.015
Average root‐mean‐square deviation of atomic coordinates (Å)^b^	
Backbone heavy atoms	0.57 ± 0.05
All heavy atoms	1.07 ± 0.06
Ramachandran analysis (%)	
Most favorable regions	85.9 ± 2.2
Additional allowed regions	12.3 ± 1.9
Generously allowed regions	1.4 ± 0.6
Disallowed regions	0.4 ± 0.4

^a^ No individual member of the ensemble exhibited distance violations > 0.5 Å or dihedral angle violations > 5 °.

^b^ Average root‐mean‐square deviation (r.m.s.d.) of atomic coordinates are calculated for individual structures with respect to the mean structure excluding ill‐defined regions (r.m.s.d. > 1.4 Å), i.e., residues 108–115, 184, 185, 201–204 and the flexible C‐terminal tail (residues 259–273).

The experimental NMR structure of the cFBR is very similar to the AF‐predicted fold [33, 34], with a backbone (N, Cα and C′) atomic r.m.s.d. value of 1.25 Å for the confidently modeled regions (Fig. 3B). However, differences are observed in the positively charged loop containing R203 and in the loop connecting the last two *β*‐strands around V241 (Figs 3B,C and S3A–C). The presence of conformational differences between the experimental NMR and AF models are confirmed unambiguously by NOESY data (Fig. S3D). For example, medium sized NOE peaks are observed between the N202 H*β* and I205Hδ resonances (these atoms are separated by 3.7 Å in the NMR structure), the V243 and S236 HN resonances (these atoms are separated by 3.1 Å in the NMR structure), and the V243 and N237 HN resonances (these atoms are separated by 3.6 Å in the NMR structure). No such NOEs are expected from the AF‐predicted structure since much longer inter‐proton distances (7.1–10.6 Å) are seen (Table S1). These differences arise primarily from local conformational differences in the loops. Note the R203 sidechain in both the NMR and AF models (Fig. 3B) is pointing away from the FBR structural domain (> 8 Å) without exhibiting any close contacts.

### The R203W substitution induces dimerization of the FBR

In contrast to both the cFBR and cFBR294, whose ^1^H^15^N HSQC spectra exhibited excellent quality without signs of heterogeneity (compared in Fig. 5), spectra of the corresponding R203W substituted domains, cR203W and cR203W294, respectively, display heterogeneity as evidenced by resonance doubling. For several resonances, one set of peaks possesses narrow line‐widths at frequency positions close to those of the unsubstituted sequence protein. A second set exhibits larger line‐widths and is located at different spectral frequencies (Fig. 4A). Such doubling of resonances suggests the presence of a second protein species in cR203W samples most likely caused by oligomerization. Therefore, ^1^H^15^N HSQC spectra of these protein constructs were recorded at multiple protein concentrations (Fig. 4B). Peak doubling persisted throughout the series, and the intensity of the broader line species increased with increasing protein concentration (Fig. 4B). TROSY‐type 3D HNCACB and HN(CO)CACB spectra recorded for a ^2^H^13^C^15^N cR203W294 sample were used to obtain backbone and C*β* assignments for the doubled peaks and confirmed that the two sets of resonances were associated with the same amino acid in the protein sequence. Since several doubled resonance sets are available in the spectra, it was possible to measure the intensities (peak volumes) of well‐resolved pairs and calculate the disassociation constant for oligomerization. The *K*_d_ values were 134 ± 43 and 110 ± 37 μm for cR203W and cR203W294 respectively.

**Fig. 4:**
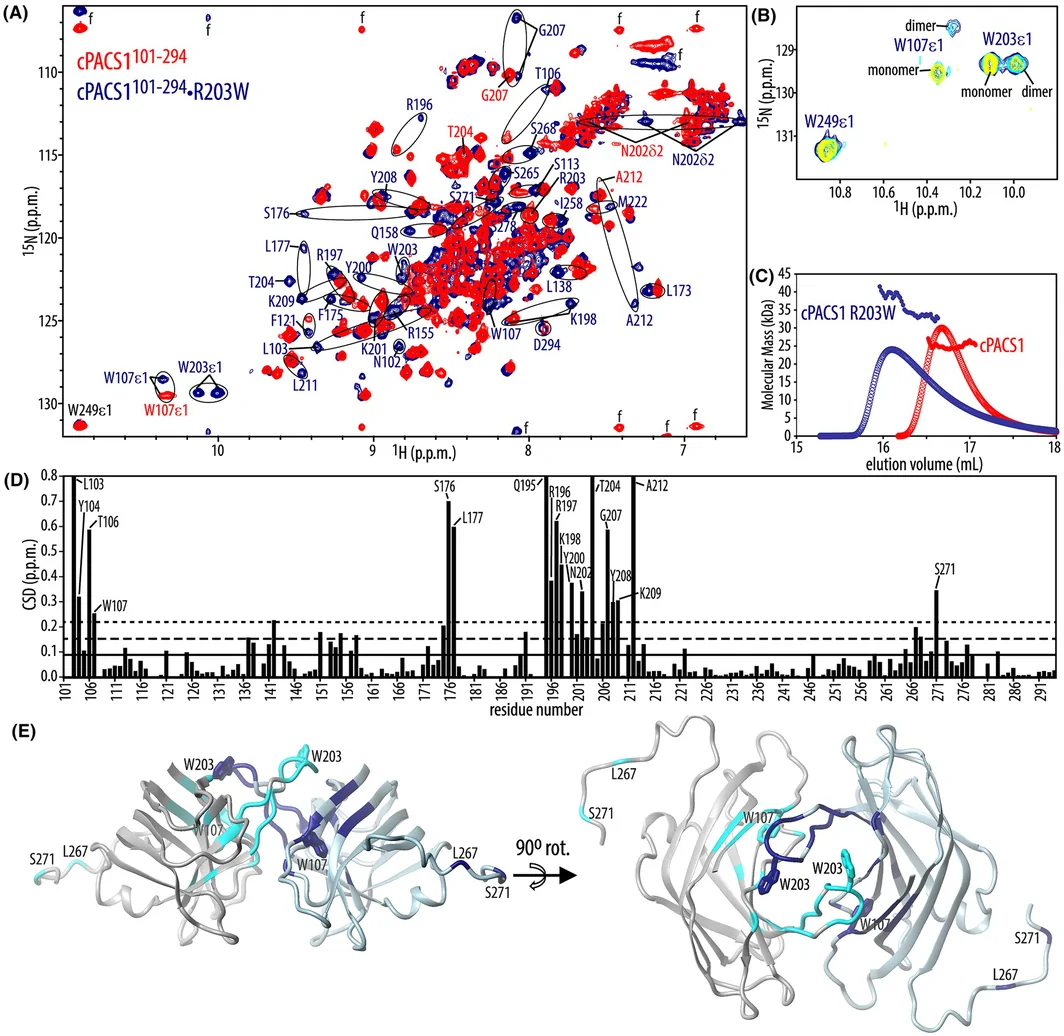
The R203W substitution induces dimerization of PACS‐1cR203W. (A) Superposition of 700 MHz ^1^H^15^N HSQC spectra of 300 μm samples of ^2^H^13^C^15^N cFBR294 (green) and ^2^H^13^C^15^N cR203W294 (navy), recorded at 30 °C and under identical buffer conditions. Resonances displaying large chemical shift differences (CSDs) are encircled in ovals and their assignments are provided by residue name and number. The letter ‘f’ was used to denote folded resonances in the ^15^N dimension. (B) Superposition of the 700 MHz ^1^H^15^N HSQC spectra of ^13^C^15^N cR203W at concentrations of 50 μm (yellow), 150 μm (cyan) and 300 μm (navy), at 30 °C and for identical buffer conditions. (C) SEC‐MALS data of cFBR294 and cR203W294. Elution profiles of cFBR294 (red) and cR203W294 (navy) are shown by open circles and the weight‐averaged molecular masses obtained from the SEC‐MALS measurements are shown by closed circles. Note, data for cR203W294 exhibit a sloping line for the molecular mass, indicative of averaging between dimer and monomer states. (D) Bar graph depicting ^1^H^15^N‐combined CSD values for monomer and dimer resonances in the cR203W294 spectrum (in A) along the polypeptide sequence. The CSD values were calculated using the equation, (Δ*δ*_*HN*_^2^ + (Δ*δ*_*N*_ */6*)^2^)^1/2^, with Δ*δ*_*HN*_ and Δ*δ*_*N*_ representing the ^1^HN and ^15^N chemical shift differences between resonance frequencies of the monomer and dimer. The CSD values of 0.090 (average), $0.153\;\left(\text{average}+\frac{1}{2}\text{stdev}\right)$ and $0.217\;p.p.m.\left(\text{average}+1\text{stdev}\right)$ are marked with solid, dashed and dotted lines, respectively. CSD values > 0.8 p.p.m. were omitted from the calculations of the average and Stdev. Some of the largest CSD values are labeled with residue names and numbers. (E) HADDOCK [38, 39] model of the cR203W dimer with the structure generated using MOLMOL v.2 K.2 [37]. The dimer structure was modeled, defining the FBR residues with large CSD values (> 0.153 p.p.m.) as active residues colored in navy and cyan, respectively, for each monomer unit.

To ascertain the oligomerization state of the R203W substituted FBRs, tandem size exclusion multi‐angle light scattering (SEC‐MALS) experiments were performed. The expected monomeric molecular mass for cFBR294 and cR203W294 is ~23 kDa. The SEC‐MALS extracted average molecular mass for cFBR294 at an injection concentration of ~140 μm was 25 kDa indicating that the unsubstituted FBR exists as a monomer. However, the average extracted mass for cR203W294 was 36 kDa with the scattering points sloping downwards across the peak from 42 to 32 kDa indicating that the substituted variant exists in a monomer/dimer equilibrium (Fig. 4C).

The dimerization interface, deduced from the amino acid identity of the doubled resonances, comprises four clusters along the linear amino acids sequence (Fig. 4D). These four regions reside on one face of the NMR structure where the basic loop and strand *β*1 are located. We generated a model for the cR203W294 dimer by docking two NMR structures onto each other using HADDOCK (Fig. 4E) [38, 39]. Interestingly, two resonances, L267 and S271, that are located in the random‐coil C‐terminal tail exhibit doubled peaks and are located on the opposite side of the FBR, away from the dimerization interface. This suggests that this random coil region, comprised of the MR, transiently contacts the dimerization interface.

### Electrostatic interactions are critical for PACS protein intramolecular regulation

Scott *et al*. reported that an acidic cluster in the MR, which can be phosphorylated by CK2 and is located just C‐terminus to the FBR, can interact with the FBR, and by doing so, regulate the interaction between PACS‐1 and various partner proteins [19]. The binding site(s) on the FBR for this regulatory acidic cluster has not been identified, but a likely candidate may be the (^196^RRKRYKNR^203^) basic cluster that contains the R203W substitution. Our above dimerization data imply that regions C‐terminus to the FBR might contact this part of the protein. To identify which regions in the PACS‐1 FBR interact with the MR, we compared the ^1^H^15^N HSQC spectra of the cFBR, which contains the basic cluster, and the cFBR294, which contains the basic cluster as well as the MR acidic cluster (Fig. 5). Chemical shift differences (CSDs) are observed for resonances of amino acids comprising the R203W induced dimerization interface, both in/near the basic loop (R197, Y200, K201, N202, R203, I205, L206) as well as surrounding components (T106 (*β*1), W107, V142 (*β*3), L177, Q178), and in a region from R152‐R155 that is outside of the dimerization interface but adjacent to the basic loop in our NMR structure. In addition, CSDs are observed for resonances associated with the C‐terminus (H260, K264, S265, L267) immediately proximal to the addition of the MR acidic cluster (Fig. 5B). Essentially, the regulatory component of the MR transiently contacts the FBR, ‘laying’ across one face of the domain to reach the basic cluster on the opposite side of the domain (Fig. 5A inset). To examine whether the electrostatic interactions between the FBR basic cluster and the MR acidic cluster are sufficient to inhibit the interaction of the PACS‐1 FBR with a partner, ^1^H^15^N HSQC spectra of ^15^N‐cFBR294 were collected in the presence and absence of na‐HDAC6 CD1. The cFBR294 ^1^H^15^N HSQC spectrum remained unchanged in the presence of HDAC6 CD1 (Fig. 6A), different from what was observed for the FBR and cFBR spectra in the presence of CD1, which exhibited a large degree of spectral broadening (compare Fig. 6A with Fig. 1A,C). Together, these data suggest that electrostatic interactions between the MR acidic cluster and the FBR basic cluster facilitate the MR folding back across the FBR, thereby regulating PACS‐1 protein–protein interactions, supporting the data of Scott *et al*. [19].

**Fig. 5:**
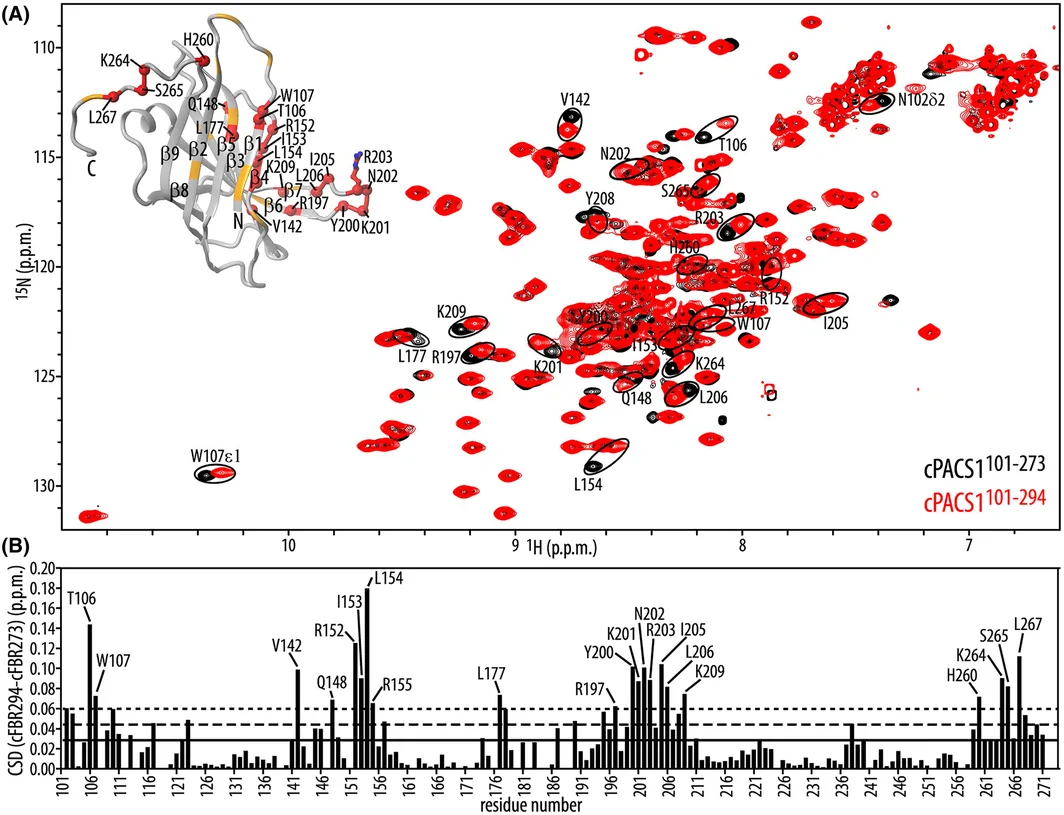
PACS‐1 MR induces chemical shifts in the basic loop containing R203. (A) Superimposition of the 800 MHz ^1^H^15^N HSQC spectra of 600 μm ^15^N cFBR (black contours) and ^15^N cFBR294 (red contours) at 30 °C under identical buffer conditions. Resonances displaying large CSD values are enclosed in ovals and assignments are provided by residue name and number. The insets depict the representative, closest to mean with lowest energy, NMR structure conformer, in ribbon representation, colored according the CSD values: red (> 0.060 p.p.m.), orange (between 0.044 and 0.060 p.p.m.) and gray (< 0.044 p.p.m.). The CSD values between the cFBR and cFBR294 were calculated using the same equation as in Fig. 4D. (B) Bar graph of the CSD values between the cFBR and cFBR294 (in A) versus residue numbers (B). The CSD values of 0.028 (average), $0.044\;\left(\text{average}+1/2\text{Stdev}\right)$ and $0.060\;p.p.m.\left(\text{average}+1S\text{tdev}\right)$ are marked with solid, dashed and dotted lines, respectively. The residues with CSD values (> 0.060 p.p.m.) are labeled with residue name and number.

**Fig. 6:**
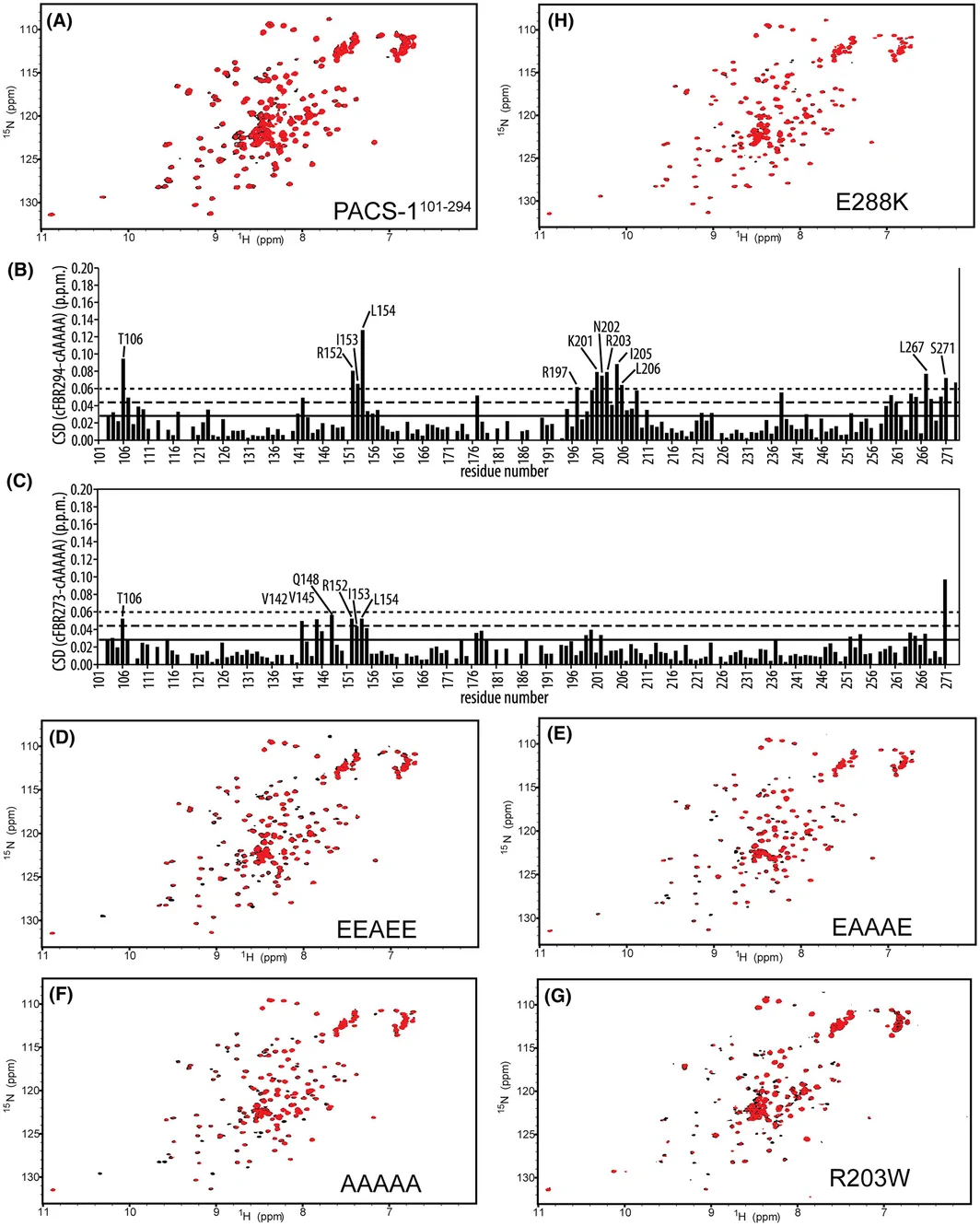
Disruption of FBR basic loop/MR acidic cluster interactions promotes PACS‐1 HDAC6 interaction. (A) ^1^H^15^N HSQC spectra of wild‐type PACS‐1 cFBR294, which contains the FBR as well as the MR, in the absence (black) or presence (red) of the HDAC6 catalytic domain (CD) 1. (B) Bar graph plots of CSD values for cFBR294 and cFBR294/AAAAA and for (**C**) cFBR and cFBR294/AAAAA versus residue numbers. The CSD values of 0.028 (average), $0.044\;\left(\text{average}+1/2\text{Stdev}\right)$ and $0.060\;p.p.m.\left(\text{average}+1S\text{tdev}\right)$ are marked with solid, dashed and dotted lines, respectively. The residues with CSD values (> 0.060 p.p.m.) are labeled with residue name and number. (D–H) ^1^H^15^N HSQC spectra of substituted cFBR294s in the absence (black) or presence (red) of the HDAC6 CD1: (D–F) cFBR294 with acidic cluster alanine substitutions in the MR; (D) EEAEE, (E) EAAAE, (F) AAAAA, (G) cFBR294 with the R203W substitution, and (H) cFBR294 with the PACS‐2 syndrome comparable substitution E288K. All ^1^H^15^N HSQC spectra were recorded at 800 MHz and 30 °C under identical buffer conditions.

To confirm that electrostatics play an important role in PACS‐1 protein–protein interactions, glutamic acids located within the MR acidic cluster, ^279^EEEEE^283^, were replaced by alanines. Variants, EEAEE, EAAAE, and AAAAA, were generated and assessed by NMR for any structural impact (Figs 6B,C and S4) and for their ability to interact with HDAC6 CD1 (Fig. 6D–F). ^1^H^15^N HSQC spectra of ^15^N‐alanine substituted variants were collected and compared to the spectrum of wild‐type cFBR294 (Fig. S4). This analysis identified three key findings. First, CSDs between the wild‐type cFBR294 and the alanine variants are restricted to FBR resonances that were responsive to the presence of the wild‐type MR (Compare Figs 5B and 6B), demonstrating that the alanine substitutions do not create new contacts between the MR and the FBR. Second, increasing the number of alanine substitutions causes resonance positions to shift toward the position of those observed in the absence of the MR (Fig. S4). Finally, when the cFBR ^1^H^15^N HSQC spectrum is compared with that of the AAAAA cFBR294 (Figs 6C and S4D), CSDs between resonances of amino acids in the basic region are substantially reduced, while those associated with other parts of the FBR, specifically T106 and V142‐ L154, remain (Fig. 6C), indicating that the ^279^EEEEE^283^ patch cannot represent the sole determinant in the FBR/MR interaction. This aligns with reports that charge reversal substitutions E209K [26] and E211K [27] in PACS‐2, which are located outside of the acidic cluster in a CK2‐phosphorylatable region, result in enhanced interaction between the PACS‐1 paralog and some of its partners. Thus, other negative charges in the cFBR294 located in the corresponding region such as E288, E290, D293, and D294 might also contribute to the electrostatic interaction or may compensate for the loss of the ^279^EEEEE^283^ patch.

We also assessed whether any of the alanine substitution variants restored interaction with HDAC6 CD 1. ^1^H^15^N HSQC spectra of ^15^N EEAEE, EAAAE, and AAAAA‐substituted cFBR294 were recorded in the absence and presence of na‐HDAC6 CD1. Resonance broadening was observed in all variants' spectra (Fig. 6D–F) indicating that removal of negative charges from the acidic cluster partially restored the ability of the cFBR294 to interact with HDAC6 CD1, but did not prevent the MR from binding the FBR basic cluster (Fig. 6D–F). Interestingly, the affinity of the alanine substituted cFBR294s for CD1, as judged by the number or resonances experiencing chemical shift broadening, did not linearly correlate with the number of substitutions. The AAAAA substituted cFBR294 ^1^H^15^N HSQC spectrum exhibited the largest number of broadened resonances (40), while the EEAEE and EAAAE‐substituted cFBR294 proteins exhibited comparable numbers (29 and 28 resonances, respectively; Fig. 6D–F). Together these data suggest that any change in the FBR/MR interaction may lead to aberrant PACS‐1/partner interaction.

The experimental NMR PACS‐1 structure (Fig. 3A) shows that the R203W substitution resides in the middle of a protruding loop that contains the basic cluster residues. Therefore, this substitution may disrupt the regulatory electrostatic interactions as well as induce hydrophobic protein–protein interactions exemplified by the observed dimerization of cR203W (Fig. 4). To examine this, ^1^H^15^N HSQC spectra of ^15^N‐cR203W294 were collected in the absence and presence of na‐HDAC6 CD1. Indeed, the observed resonance broadening indicates that the R203W substitution allowed the cFBR294 to interact with CD1 (Fig. 6G).

Charge reversal substitutions of glutamic acids neighboring the MR acidic cluster in PACS‐2 enhance interactions with partner proteins [26]. Therefore, we evaluated whether a charge reversal in the MR is sufficient to disrupt the intramolecular regulation of PACS proteins. The corresponding PACS‐2 E209K substitution, E288K, was introduced into the PACS‐1 cFBR294, and ^1^H^15^N HSQC spectra of ^15^N protein were collected in the absence and presence of na‐HDAC6 CD1. Although we observed some line broadening for a few resonances, the E288K substitution did not restore the PACS‐1/HDAC6 interaction to the same degree as any of the alanine substitutions in the basic cluster or the R203W syndrome‐causing substitution (Fig. 6H).

Unlike the 88% amino acid identity between the PACS‐1 and PACS‐2 FBRs, the MR, proximal to the FBR, exhibits more divergence and only possesses 58% identity. The PACS‐2 acidic cluster is notably disrupted by a tyrosine (PACS‐1 ^279^EEEEE^283^: PACS‐2 ^200^EEEYE^207^), which may result in other negative charges in the PACS‐2 MR having a greater contribution to the electrostatic interactions between the FBR and MR (Fig. 7A). To evaluate this possibility, ^1^H^15^N HSQC spectra of ^15^N‐PACS‐2^22‐213^, the equivalent of the PACS1 cFBR294, with and without the E209K substitution were recorded in the absence and presence of the na‐SIRT1 N‐terminus (SIRT1^1‐233^). PACS‐2 recognizes the SIRT1 N‐terminus through a bipartite binding site [22, 40]. Contrary to the PACS‐1/HDAC6 interaction, the presence of the PACS‐2 MR did not completely abrogate interaction with SIRT1 (Fig. 7B, C). However, the E209K variant exhibited more extensive resonance broadening indicating that it was less efficient at blocking the interaction (Fig. 7D). Since NMR studies necessitate relatively high concentrations of components, we posited that more pronounced effects may be observable under more dilute conditions. To this end, we supplemented our NMR studies with analytical gel filtration experiments. The SIRT1 N‐terminus eluted earlier from the column than PACS‐2 due to its unstructured nature despite comparable molecular masses (25 and 22 kDa respectively). Whether injected alone or in the presence of the SIRT1 N‐terminus, wild‐type PACS‐2^22‐213^ eluted at the same position with only a minor decrease in peak intensity. However, the PACS‐2^22‐213^E209K peak intensity decreased by 50% in the presence of SIRT1 with an intensity increase in the earlier eluting peak, where the complex also elutes (Fig. 7E). These data indicate that the negatively charged amino acids neighboring the MR acidic cluster also contribute to PACS protein intramolecular interaction.

**Fig. 7:**
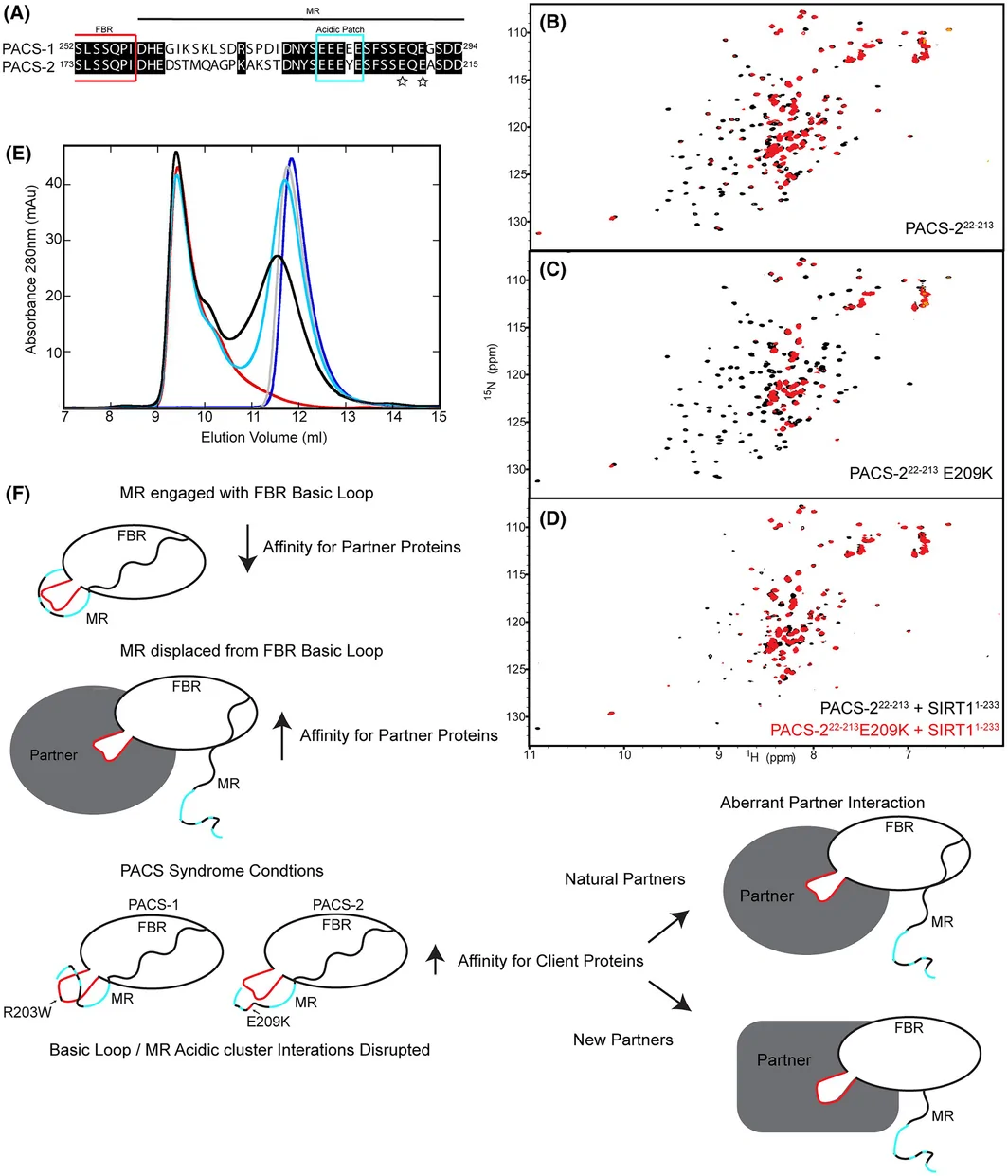
PACS‐2 syndrome causing E209K substitution promotes PACS‐2/SIRT1 interaction. (A) Amino acid sequence comparison (ClustalX2 [41]) of the MR region in *Homo sapiens* PACS‐1 (Q6VY07) and PACS‐2 (Q86VP3) used in this study. The end of the structured FBR is marked in a red box. The MR acidic cluster is enclosed in a cyan box. PACS‐2 syndrome disease‐causing substitutions E209K and E211K are marked with stars. ^1^H^15^N HSQC spectra of 30 μm (B) PACS‐2^22−213^ or (C) PACS‐2^22−213^ E209K (the PACS‐2 equivalent of the PACS‐1 cFBR294) in the absence (black) or presence (red) of 30 μm of the SIRT1 N‐terminus (SIRT1^1−233^). (D) Superposition of the ^1^H^15^N HSQC spectra of PACS‐2^22−213^/SIRT1^1−233^ (black) and E209K/SIRT1^1−233^ (red). (E) Gel filtration A_280_ profiles of SIRT1^1−233^ (red), PACS2^22−213^ (blue), PACS2^22−213^ in the presence of SIRT1^1−233^ (cyan), PACS2^22−213^E209K (gray), and PACS2^22−213^E209K in the presence of SIRT1^1−233^ (black). (F) Cartoon representation of the PACS protein FBR/MR interaction. Negatively charged amino acids (cyan) in the MR engage in electrostatic interactions with the basic loop (red) of the FBR lowering the affinity of PACS proteins for their partners. Disruption of the electrostatic interactions increases the affinity of PACS proteins for its partners. Substitutions in either the basic loop (as associated with PACS‐1 syndrome) or the MR (as associated with PACS‐2 syndrome) alter these interactions and lead to a higher basal affinity for partner proteins. Furthermore, substitution of hydrophobic residues for charged residues may lead to interaction with non‐canonical partners.

## Discussion

The genetics behind disease manifestation are complex and poorly understood. This is supported by numerous GWAS that have examined thousands of genes to identify those strongly linked with specific diseases [13, 42, 43]. However, pinpointing causal variants remains challenging due to unknown mechanisms of action, linkage disequilibrium and small patient sample sizes [44, 45]. About 300 individuals with PACS‐1 syndrome have been identified with a few thousand predicted to exist worldwide [46]. The enzyme, HDAC6, has been identified as an effector for the pathogenesis associated with the PACS‐1 R203W substitution [10]. Its hyperactivity correlates with increased association with PACS‐1 R203W and causes significant disruption of the cytoskeleton and Golgi apparatus in neural cells, leading to several neural deficits *in vivo* [10]. Here, we provide direct physical evidence for the increased binding between the PACS‐1 R203W variant and HDAC6. The MR of PACS‐1 has been reported to modulate protein–protein interactions by interacting with the FBR, thereby preventing this domain from engaging in such interactions [19]. We structurally investigated this notion by determining the solution structure of the FBR (Fig. 3) and demonstrated that the N‐terminus of the MR interacts with one face of the FBR including the large positively charged loop on the opposite side of the domain (Fig. 5). The interaction of the MR acidic cluster with the basic loop was sufficient to block the interaction between the PACS‐1 FBR and HDAC6 CD1 (Fig. 6). These regulatory electrostatic contacts can be disrupted by removal of charged residues in either the FBR basic cluster or the MR acidic cluster. With respect to PACS‐1 syndrome, we specifically demonstrated that the R203W substitution in PACS‐1 permits the PACS‐1/HDAC6 interaction in the presence of the regulatory MR acidic cluster (Fig. 5). These findings suggest that PACS‐1 syndrome, which involves increased PACS‐1/HDAC6 interaction, results from a loss of an intramolecular autoregulatory interaction within the PACS‐1 protein.

Despite severe cellular consequences resulting from cytoskeletal disruption and Golgi dispersal, a surprising aspect of PACS‐1 syndrome is that the clinical features vary [9, 15, 16, 17, 47, 48, 49, 50], strongly suggesting that the PACS‐1/HDAC6 interaction is not the sole contributor to disease manifestation. PACS‐1, by virtue of its activity as a cellular cargo sorting protein [1], interacts with numerous partners [51], suggesting that its dysregulation might result in a multitude of effects. Our NMR analysis of cR203W revealed a substitution‐induced dimerization of the PACS‐1 FBR. The observed dimerization constant (*K*_d_ ~ 150 μm) reflects weak association, and a high ‘local effective concentration’ would be required if oligomerization were a pathogenic feature of the R203W substitution. Irrespective, the observed dimerization reveals that this pathogenic substitution allows PACS‐1 to interact with non‐canonical partners. Therefore, considering the diversity of the human genome, it is reasonable to suggest that the spectrum of clinical features experienced by PACS‐1 syndrome afflicted individuals, while primarily a manifestation of the PACS‐1/HDAC6 interaction, is a result of multiple PACS‐1/wild‐type partner and PACS‐1/R203W‐induced partner interactions specific to an individual's genetic makeup.

Our work regarding the R203W substitution in PACS‐1 is not only relevant for PACS‐1 syndrome. The human genome contains two *PACS* genes encoding PACS‐1 and PACS‐2. Mutations in both genes are associated with neurodevelopmental disorders exhibiting several overlapping symptoms. While the PACS‐1 pathogenic substitution is located in the basic loop of the FBR, the PACS‐2 pathogenic substitutions, E209K and E211K, are located just beyond the MR acidic cluster (^200^EEEYE^204^) in a region undergoing phosphorylation [26, 27]. We hypothesized that these additional charged residues also contribute to changes in intramolecular interactions for two reasons. In the original report by Olson *et al*. [26], enhanced immunoprecipitation was observed between the E209K‐substituted PACS‐2 and three partner proteins. Also, we observed that removing all negative charges from the PACS‐1 MR acidic cluster did not prevent the MR/FBR interaction (Figs 6 and S2). Indeed, in the PACS‐2 context, the E209K containing protein interacted more tightly with one of its partners, SIRT1. In contrast, the introduction of the equivalent change, E288K, into PACS‐1 did not affect the PACS‐1/HDAC6 interaction, as evidenced by our data for the alanine substitutions in the acidic cluster. Whether this is associated with the presence of a tyrosine in place of one of the glutamates in the PACS‐2 acidic cluster needs further investigation. Irrespectively, both PACS‐1 and PACS‐2 disease‐associated genetic changes result in PACS proteins in which the intramolecular interaction between the FBR basic loop and the acidic residues in the N‐terminus of the MR are perturbed.

While this manuscript demonstrates that the negatively charged amino acids in the MR, proximal to the FBR, are critical to PACS protein intramolecular regulation, phosphorylation of the serines in this region has previously been shown to play a critical role in PACS/partner interaction [19, 52]. Phosphorylation of S278 in PACS‐1, located immediately prior to the acidic cluster, enhances the PACS‐1/Nef interaction [19], while phosphorylation of S207, S208, and S213 in PACS‐2, located immediately C‐terminal to the acidic cluster, is critical for PACS‐2 association with the PKD2 (polycystic kidney disease 2) ion channel and their combined localization to mitochondria‐associated ER membranes (MAMs) [52]. Based on the findings in this manuscript, one would predict that serine phosphorylation in the MR, proximal to the acidic cluster, would repress PACS protein–protein interactions since the additional negative charge(s) would enhance electrostatic interactions with the FBR basic cluster. However, this is not the case. Therefore, PACS proteins likely rely on a third party that specifically recognizes the phosphoserine while being indifferent to the negative charge of an acidic cluster, such as an FHA (Forkhead‐Associated) domain [53], WW (tryptophan‐tryptophan) domain [54], or a BRCT (BRCA1 C‐terminus) domain [55]. Targeting the phosphoserine followed by subsequent interaction with the acidic cluster could act as a mechanism to free the basic loop and initiate PACS/partner interaction.

Finally, our work suggests a mechanism by which PACS proteins sample the proteome and discern their diverse partners. In multiple instances, PACS proteins recognize a bipartite binding site on their partner that is composed of an acidic cluster and hydrophobic residues in an α‐helix [21, 22] with the α‐helical amino acids comprising the higher affinity site [22]. In this manuscript, we demonstrated that the loop containing most of the basic cluster needs to be freed from intramolecular interaction with the MR to engage a partner, such as HDAC6. Our NMR structure identified this loop as ^199^ **R**Y**K**N**R**TIL^206^. In the wild‐type sequence, positive charge essentially extends over the length of the loop apart from the last three amino acids, making electrostatics the primary mechanism for this loop to engage a partner. However, in the R203W substitution (^199^ **R**Y**K**NWTIL^206^), the charges on the surface are reduced, with hydrophobic side chains becoming available for interaction and forming the dimerization interface (Fig. 4E). We propose that the basic loop probes for acidic clusters, sampling prospective partners, with a separate part of the FBR governing selectivity. Notably, the solution structure indicates that partner selectivity is not determined by a single region of the FBR. Mapping residues important for the PACS‐1/GGA3 interaction ^249^KIY^251^ [20] and the PACS‐1/Nef interaction ^181^HFLKRDANKLQIMLQ^195^ [21] onto our structure indicates that these regions lie on opposite sides of the *β*‐core.

## Conclusion

Our structural analysis of the PACS‐1 FBR reveals that PACS‐1 protein–protein interactions are regulated through electrostatic interactions between a basic loop in the FBR and an acidic and post‐translationally modified part of the MR. Substitutions in these regions, such as the PACS‐1 syndrome causing R203W substitution, disrupt the electrostatic‐based intramolecular regulation and drive aberrant protein–protein interactions (Fig. 7F).

## Materials and methods

### Cloning and protein expression

The cloning and protein expression of PACS‐2 FBR^22‐197^ were described previously [22]. PACS protein constructs unique to this study, PACS‐1 FBR^101‐273^, PACS‐1 cFBR294^101‐294^, and PACS‐2^22‐213^ were inserted into a modified pET41 vector using Gibson assembly, expressed, and purified, essentially as reported previously [22]. All protein variants were generated using non‐overlapping site‐directed mutagenesis. Briefly, all DNA constructs were transformed into a custom‐made *E. coli* strain termed Rosetta* cells [22]. For isotopically (^13^C, ^15^N) enriched proteins, cells were grown at 37 °C in modified M9 medium, supplemented with ^15^N‐ammonium chloride or ^15^N‐ammonium chloride and ^13^C‐glucose until OD_600_ = 0.6. Protein expression was induced with 100 μm IPTG and cultures were grown overnight at 18 °C for 16–18 h. Cells were harvested by centrifugation (4600 ***g***/10 min/4 °C) and resuspended in 20 mm HEPES pH 7.5, 150 mm NaCl, 0.5 mm TCEP for lysis using a microfluidizer (Microfluidics, Westwood, MA, USA). The lysate was clarified by centrifugation (38 000 ***g***/30 min/4 °C) and applied to a SP Sepharose column (Cytiva; Marlborough, MA, USA). Bound protein was eluted with a 300–700 mm NaCl gradient. Protein‐containing fractions were tandemly buffer exchanged and separated from any trace contaminants with a final gel filtration step using a Superdex 75 16 60 column (Cytiva, USA).

The *D. rerio* HDAC6 CD1^55‐419^ and CD2^440‐798^ were cloned into a modified pET41 vector using Gibson assembly. The modified vector was a pET41 backbone in which everything between the NdeI and XhoI sites was replaced with the following NdeI‐KSS‐6xHistag‐GSS‐MBP‐NGSTGGSTGGT‐KpnI‐TEV‐EcoRI‐XhoI. HDAC6 CD1^55‐419^ Gibson assembly resulted in the elimination of the EcoRI site such that the only cloning artifact following TEV cleavage is a single N‐terminal serine. HDAC6 CD2^440‐798^ Gibson assembly likewise resulted in the elimination of the EcoRI site but introduced a GGS tripeptide between the final serine in the TEV site and the serine at amino acid position 440. This SGGS addition was necessary for efficient TEV cleavage. Both DNA constructs were transformed into the Rosetta* cells [22] and protein was expressed using autoinduction media. Briefly, a 50 mL starter culture containing non‐inducing glucose was grown overnight. The next day 5 mL of overnight were pelleted (3200 ***g***/10 min/25 °C) and resuspended in 500 mL of autoinduction medium in a 2 L baffled flask. Cells were grown at 37 °C for 3.5 h (OD_600_ ~ 1.5) before the temperature was lowered to 18 °C for a further 16‐h growth period. Cells were harvested by centrifugation (4600 ***g***/10 min/4 °C) and resuspended in 20 mm HEPES pH 8.0, 1 m NaCl, 20 mm imidazole, 0.5 mm TCEP for lysis using a microfluidizer (Microfluidics). The lysate was clarified by centrifugation (38 000 ***g***/30 min/4 °C) and applied to a Histrap‐HP column (Cytiva, USA). Bound protein was eluted using a linear 40–500 mm imidazole gradient and the pooled fractions were subjected to gel filtration using a Superdex 200 26 60 column (Cytiva, USA) equilibrated in 20 mm HEPES pH 7.5, 150 mm NaCl, 0.5 mm TCEP. The desired peak was TEV treated at 1 : 20 (1 mg TEV: 20 mgs MBP‐HDAC6). The sample was then adjusted to 500 mm NaCl and 20 mm imidazole and rerun over the Histrap column to remove the MBP and TEV. The flow‐through containing HDAC6 was concentrated and subjected to a final gel filtration step using a Superdex 75 16 60 (Cytiva, USA) column.

For all proteins, the final gel filtration buffer was that of the reported conditions for a particular NMR experiment. Final protein purity was estimated at > 98% by SDS/PAGE.

### Generation of the PACS‐1 FBR chimera

There are 34 amino acid differences between the PACS‐1 and PACS‐2 FBRs and three of the differences are aspartic acid/glutamic acid substitutions. The remaining 31 differences were divided into five groups along the linear sequence (Fig. S2), and each group of substitutions was ordered as a gblock from IDT (Coralville, IA, USA). The gblocks were inserted into a pET41 vector between the NdeI and XhoI restriction sites [22]. Each chimeric FBR protein was expressed and purified as described above. Of the five chimeras, the one containing V241A, S242P, P244K, V245A, K249W, and Y251A was selected for further study since its ^1^H^15^N HSQC spectrum was of good quality (uniform peak shapes and intensities) and most closely represented the wild‐type FBR. Each amino acid change in this set of five was assessed as to its effect by changing the codon back to the original sequence. Reversions of V241A, S242P, V245A did not affect the quality of the spectrum, while K249W and Y251A were critical for spectral improvement. In addition, P244K enhanced the yield of the purified protein. The resultant tri‐substituted PACS‐1 FBR of P244K, K249W, and Y251A maintained all the enhanced spectral quality of the original hexa‐substituted one.

### Circular dichroism

Circular dichroism measurements were collected on a Jasco (Oklahoma City, OK, USA) J‐810 spectropolarimeter with the following settings: wavelength; 220 nm., bandwidth; 1 nm; temperature slope; 1 °C/min, data pitch; 0.2 °C; response time; 1 s; and temperature interval of 20–85 °C. A 0.1 cm cuvette was used for all samples, and the protein concentration was 50 μm in a total volume of 150 μL in 20 mm citrate pH 6.5, 70 mm NaCl.

### Multi‐angle light scattering

Size exclusion chromatography/multi‐angle light scattering (SEC‐MALS) data were collected at room temperature using a Waters (Milford, MA, USA) Alliance HPLC system interfaced with TAW e2695 separation and TAW 2489 variable UV detector modules on a Superdex 200 10 300 increase column (Cytiva, USA) with in‐line multi‐angle light scattering (HELEOS, Wyatt Technology, Santa Barbara, CA, USA) and refractive index (Optilab rEX, Wyatt Technology) detectors. 100 μL of cFBR294 or cR203W294 protein at a concentration of 3 μg/μL was loaded onto the column equilibrated with 20 mm sodium citrate buffer pH 6.5/70 mm NaCl/0.5 mm TCEP/0.02% sodium azide and were eluted at a flow rate of 0.5 mL/min. The molecular masses of eluted proteins were determined using the ASTRA v.7.1.2.5 program (Wyatt Technologies, USA).

### NMR spectroscopy

NMR spectra were recorded at 293, 300 or 303 K on a Bruker (Billerica MA, USA) AVANCE 600, 700, 800, or 900 MHz NMR spectrometer equipped with a z‐axis gradient cryoprobe, using protein samples prepared in 20 mm citrate pH 6.5/ 70 mm NaCl/0.5 mm TCEP and 7–10% or 100% D_2_O. When comparing ^1^H^15^N HSQC spectra, each comparison (i.e., each figure) began with the acquisition of the appropriate wild‐type spectrum followed by that of the specified comparative spectra. Gratifyingly, the baseline, wild‐type spectra were unchanged from aliquot to aliquot under identical conditions. For backbone and sidechain assignments, heteronuclear 3D HNCA, HN(CO)CA, either TROSY‐ or non‐TROSY‐HNCACB and HN(CO)CACB spectra were obtained using ^2^H^15^N^13^C‐labeled cFBR, cFBR294, and cR203W294. 3D HCCH‐TOCSY (with a mixing time of 10.9 ms in 100% D_2_O) and simultaneously ^13^C/^15^N‐edited NOESY (with a mixing time of 120 ms in 7% or 100% D_2_O) experiments were performed using ^15^N^13^C cFBR, cFBR294, and cR203W294. Protein concentrations of 300, 584 or 600 μm were used for the 3D heteronuclear experiments. All data were processed with NMRPipe v.9.9 [56] or TopSpin 3.2 (Bruker, USA), and analyzed with CCPN v.2.4.0 [57].

### NMR structure determination

Structures were calculated with XPLOR‐NIH v.3.6 [35]. An iterative approach with extensive manual cross‐checking of all distance restraints against the NOESY data and calculated structures was employed using CCPN v.2.4.0 [57]. The final number of NOE‐derived distance restraints was 4689. Additionally, 279 *φ* and *ψ* backbone torsion angle restraints were extracted via TALOS‐N [36] based on HN,H*α*, C*α*, C*β* and N chemical shifts. 1000 structures were generated, and the lowest 40 energy structures were used for further analysis using XPLOR‐NIH v.2.53 and PROCHECK‐NMR v.3.3 [58]. Individual conformers exhibited no distance violations > 0.5 Å and dihedral violations > 5 °. All structure figures were generated with MOLMOL v.2 K.2 [37].

### Dissociation constant determination from 2D ^1^H^15^N HSQC spectra of cR203W

Well‐resolved amide resonances for 7–8 residues associated with the monomeric and dimeric forms were selected in the ^1^H^15^N HSQC spectra of 300 μm ^13^C^15^N cR203W and ^2^H^13^C^15^N cR203W294 NMR samples. Peak volumes were measured in ccpn and *K*_d_ values were calculated using *K*_d_ = 2*P*_t_ *f*_M_^2^/*f*_D_ with *P*_t_, representing the total protein concentrations in the NMR samples, and *f*_M_ and *f*_D_, the measured fractions of monomer and dimer, respectively [59].

### Dimer modeling of the cR203W

The dimer structure was modeled using haddock v.2.4 [38, 39]. Amino acids whose resonances exhibited large chemical shift differences (CSD > $\text{average}+/-\frac{1}{2}\mathrm{STD}$) were selected as residing in the dimer interface, namely 103, 104, 106, 107, 142, 151, 155, 158, 175–177, 191, 195–198, 200–204, 206–209, and 212 (Fig. 4D). Resonances L267, S268, and S271 also showed large CSD but were excluded since they reside in the C‐terminal random coil region. The representative NMR structure of PACS1^101‐273^, after R203W substitution in PyMOL v.4.30 [60] was used as the input structure for the modeling.

## Author contributions

TCK was involved in conceptualization, investigation, writing – original draft, review and editing. ILB was involved in conceptualization, investigation, writing – original draft, review and editing. RP was involved in investigation. MEL was involved in investigation. LT was involved in investigation. CD was involved in investigation. GT was involved in conceptualization, funding acquisition, writing – review and editing, project administration. AMG was involved in conceptualization, funding acquisition, writing – review and editing, project administration, supervision.

## Conflict of interest

The authors declare no conflict of interest.

## Supporting information


**Table S1.** Interproton differences between NMR measurements and the AlphaFold prediction.
**Fig. S1.** Dependence of spectral quality on protein concentration.
**Fig. S2.** Alignment of PACS‐1 and PACS‐2 protein sequence.
**Fig. S3.** Comparison of the PACS‐1 cFBR NMR structure with the AlphaFold prediction.
**Fig. S4.** Chemical shift perturbations caused by the mutations of the acidic cluster residues in the MR.

## Data Availability

The PACS‐1 cFBR structure has been deposited in the wwPDB (ID 9Z30) and the BMRB (ID 31281).
