# Supplementary material for: The R203W substitution drives PACS‐1 syndrome by disrupting intramolecular regulation
Source: FEBS J. 2026 Mar 20;293(19):5717–34. doi: 10.1111/febs.70492 (PMC13480987; doi:10.1111/febs.70492)

**Supporting Information** contains one supplementary table (**Supplemental Table S1**) and four supporting figures (**Supplemental Fig. S1, Supplemental Fig. S2 and Supplemental Fig. S3 and Supplemental Fig. S4**).

**Supplemental Table S1**. **Inter-proton differences between NMR measurements and the AlphaFold prediction.** Examples of inter-proton distances that are significantly different in the experimental NMR and predicted AF structures, derived from the presence of the NOE peaks in 3D $^{15}N$-NOESY spectra of $^{13}C^{15}N$ cFBR at 600 μM, pH 6.5, 303K, 800 MHz (**Fig. S3D**).

**Supplemental Figure S1– Dependence of spectral quality on protein concentration.** Comparison of PACS protein $^{1}H^{15}N$ HSQC spectra at varying concentrations. The $^{1}H^{15}N$ HSQC spectra of the (**A, B**) PACS-2 FBR (PACS-2$^{22\text{-}197}$), (**C, D**) the PACS-1 FBR (PACS1$^{101\text{-}273}$), (**E, F**) the chimeric PACS-1 cFBR, and (**G, H**) the R203W substituted chimeric PACS-1 cR203W at either 30 μM (**A, C, E, G**) or 200 μM (**B, D, F, H**). Examples of spectral regions or quality at the higher protein concentration are enclosed in red symbols. Spectra of all proteins were collected at pH 6.5, 30°C, 800 MHz.

**Supplemental Figure S2–Alignment of PACS-1 and PACS-2 protein sequence.** Clustal X2 was used to align *Homo sapiens* PACS-1 (Q6VY07) and PACS-2 (Q86VP3) protein sequences. The portion of the alignment showing the N-terminus through the FBR domain is shown. The FBR is enclosed in a red box, with cyan and dark blue boxes highlighting amino acid differences within the FBR. The dark blue boxes indicate those amino acids substituted in the PACS-1 FBR from which the cFBR was derived.

**Supplemental Figure S3 – Comparison of the PACS-1 cFBR NMR structure with the AlphaFold prediction.** (**A)** The average Cα coordinate displacements (solid bars) among the final 40 conformer NMR structure ensemble with the standard deviation indicated (capped lines). (**B)** Cα coordinate displacements between the mean experimental NMR and predicted AF structures. (**C**) adjusted Cα coordinate displacements between the mean experimental NMR and predicted AF structures were obtained by subtracting the average Cα displacements (plus 1 Stdev) among the 40 conformer NMR structural ensemble (**A**) from the Cα coordinate displacements between the mean experimental NMR and predicted AF structures (**B**). (**D)** Selected strips of the 3D $^{15}N$-edited NOESY spectrum indicative of structural differences between the experimental NMR and predicted AF structures. NOE peaks that are only compatible with the experimental NMR but not the AF structure are enclosed by red circles. The 800 MHz 3D NOESY data was acquired using a sample containing 600 μM $^{13}C^{15}N$ cFBR in 20 mM citrate (pH 6.5) buffer at 303 K.

**Supplemental Figure S4 – Chemical shift perturbations caused by the mutations of the acidic cluster residues in the MR.** Superposition of 800 MHz $^{1}H^{15}N$ HSQC spectra of $^{13}C^{15}N$ cFBR294 (red) with that of (**A**) $^{15}N$ cFBR294 EEAEE (dark green), (**B**) EAAAE (light green), and (**C**) AAAAA (cyan). (**D**) Superposition of the $^{15}N$ cFBR294 AAAAA $^{1}H^{15}N$ HSQC spectrum with that of $^{13}C^{15}N$ cFBR (black). Protein concentrations of the samples ranged from 584-600 μM. Spectra were in 20 mM citrate buffer, pH 6.5 at 303 K. Resonances that exhibit chemical shift differences are labelled with the corresponding residue name and number.

**Supporting Table S1.** Inter-proton distances (Å) in the experimental NMR and predicted AF structures

| atom 1 | atom 2 | NMR[a] | AF |
|---|---|---|---|
| V243 HN | S236 HN | 3.1 | 7.1 |
| V243 HN | N237 HN | 3.6 | 7.8 |
| V241 HN | N237 HA | 4.3 | 9.1 |
| V241 HN | N237 HN | 4.1 | 8.0 |
| V241 HN | V238 HN | 4.7 | 8.9 |
| N202 HCβ | I205 HCγ2 | 6.0 | 11.7 |
| N202 HCβ | I205 HCδ1 | 3.7 | 10.6 |
| I205 HCγ2 | Y200 HCβ | 5.6 | 7.0 |
| I205 HCδ1 | Y200 HCβ | 5.4 | 8.0 |
| I205 HCδ1 | Y200 HCδ | 5.4 | 8.1 |
| K201 HCε | Y200 HCδ | 4.0 | 10.3 |
| K201 HCε | Y200 HCε | 4.9 | 12.5 |

[a]Inter-proton distances are from the representative, closest to the mean with lowest energy, NMR structure.

Supplemental Figure 1

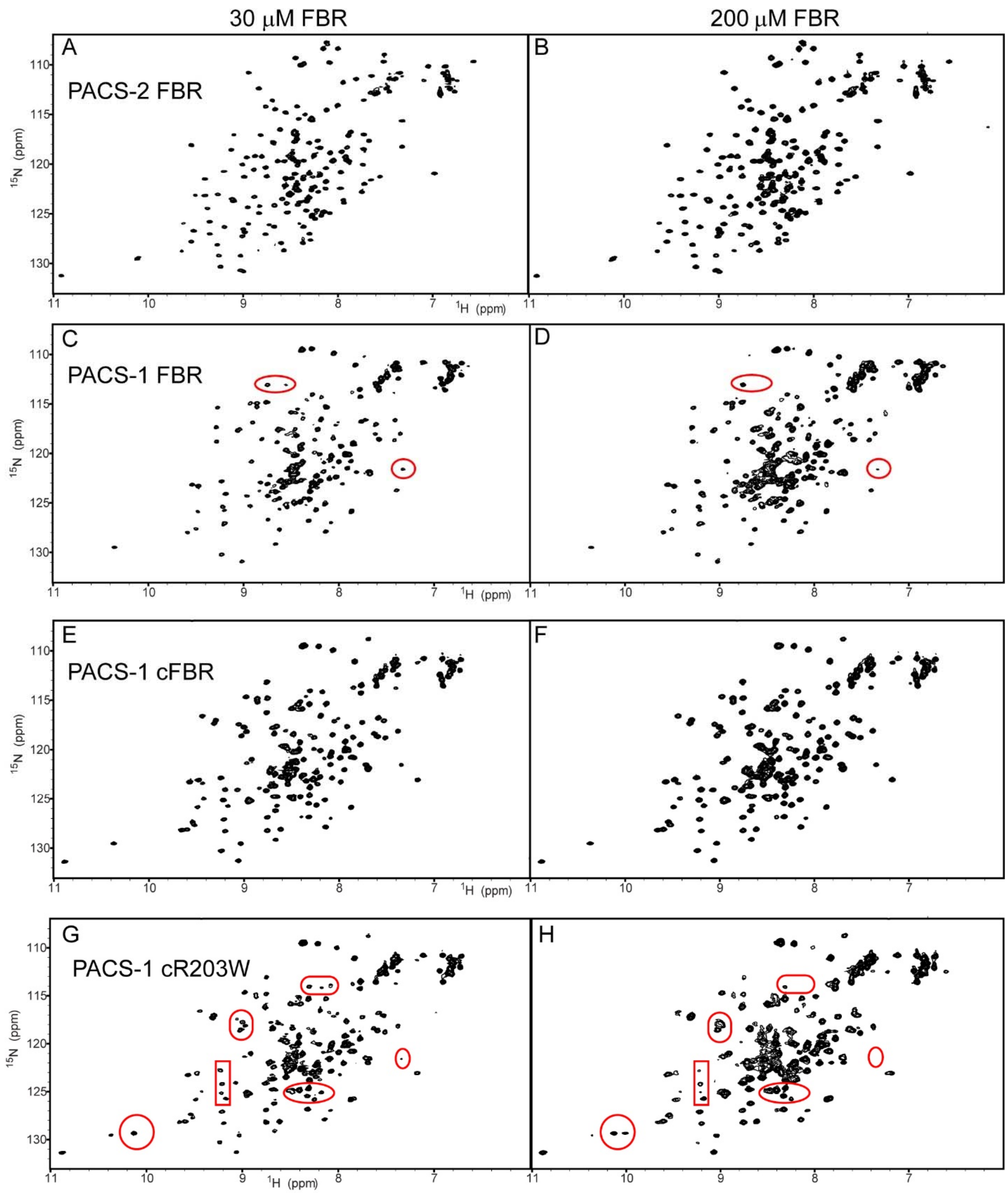

## Supplemental Figure 2

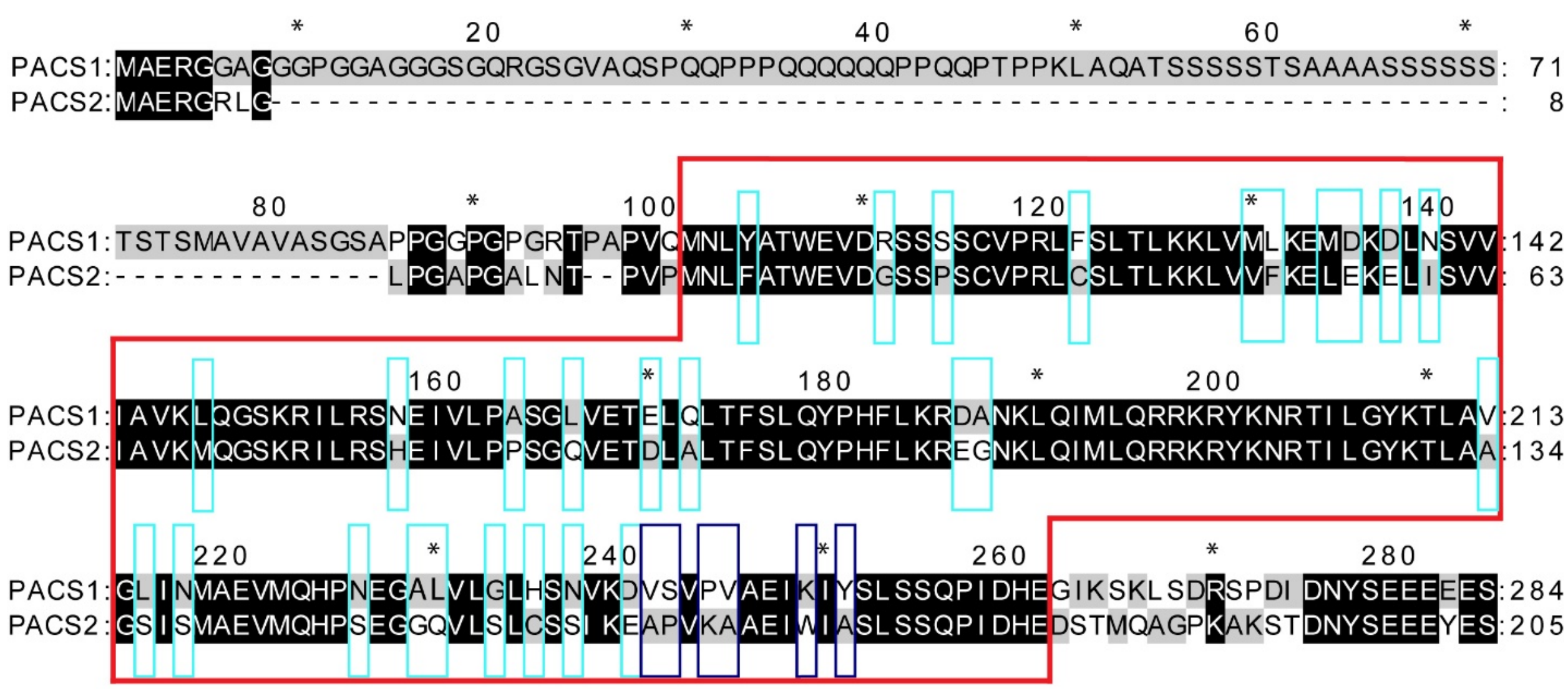

Supplemental Figure 3

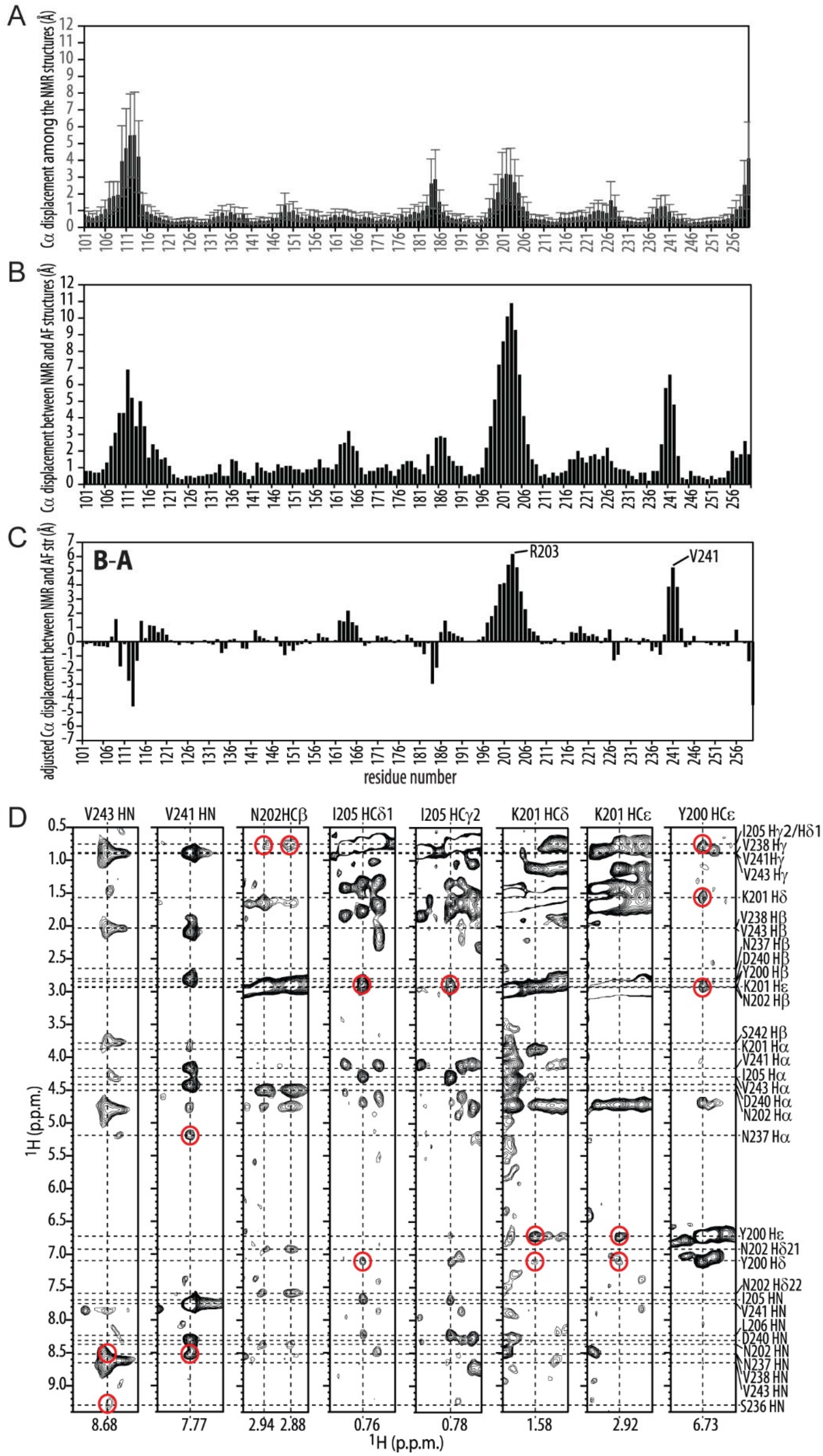

Supplemental Figure 4

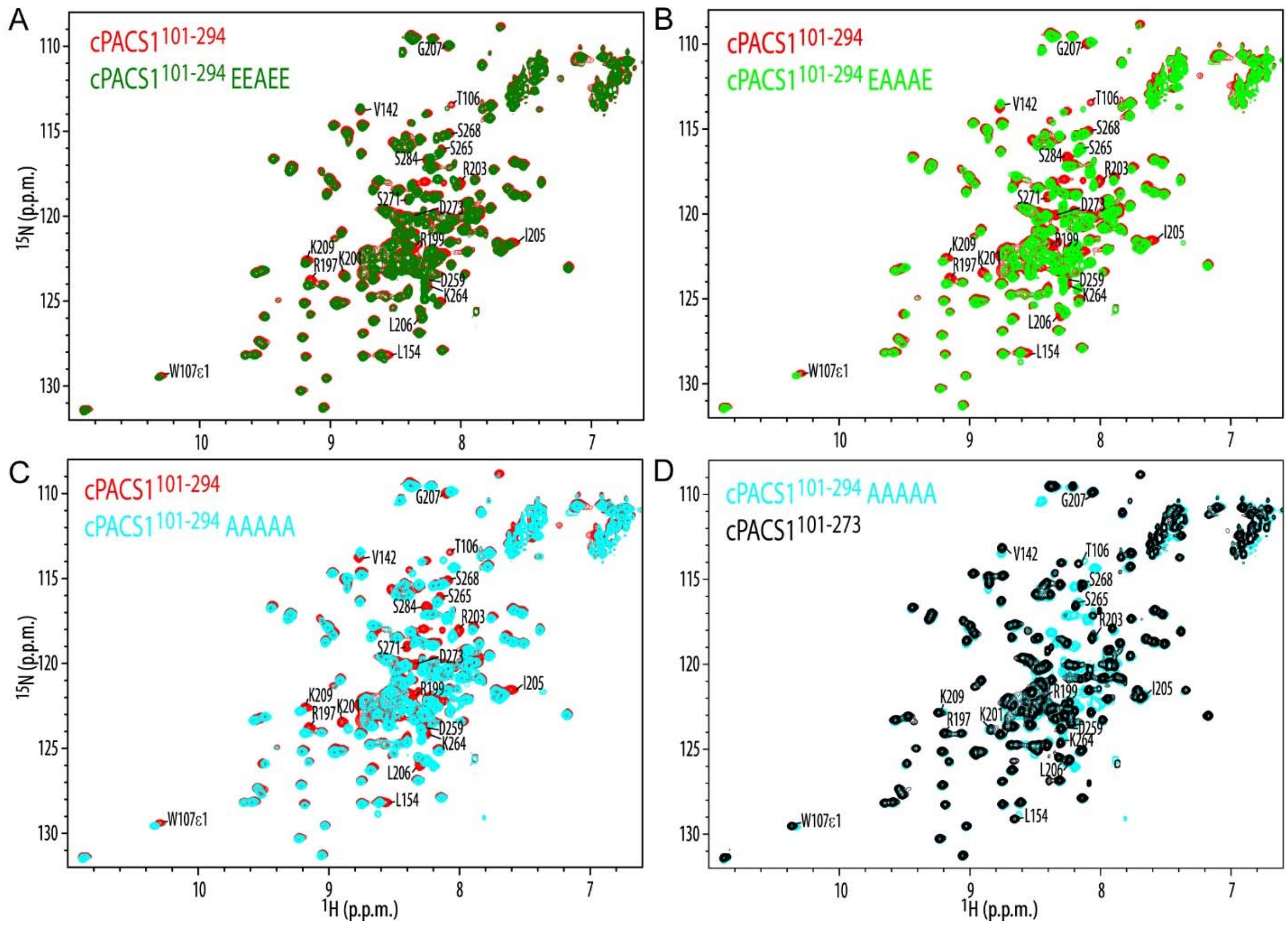

Supplement: Supplementary file 1 [file FEBS-293-5717-s001.pdf]
